# A case of appendiceal ganglioneuroma in neurofibromatosis type 1

**DOI:** 10.1186/s40792-021-01299-0

**Published:** 2021-09-28

**Authors:** Tadaaki Shimizu, Nao Hondo, Yusuke Miyagawa, Masato Kitazawa, Futoshi Muranaka, Shigeo Tokumaru, Satoshi Nakamura, Makoto Koyama, Yuta Yamamoto, Takehito Ehara, Satoru Miyazaki, Yasuhiro Iijima, Mai Iwaya, Yuji Soejima

**Affiliations:** 1 Division of Gastroenterological, Hepato-Biliary-Pancreatic, Transplantation and Pediatric Surgery, Department of Surgery, Shinshu University School of Medicine, 3-1-1 Asahi, Matsumoto, Nagano 390-8621 Japan; 2 Department of Laboratory Medicine, Shinshu University Hospital, Matsumoto, Nagano 390-8621 Japan

**Keywords:** Neurofibromatosis type 1, Appendix, Ganglioneuroma

## Abstract

**Background:**

Neurofibromatosis type 1 is an autosomal dominant inherited disease associated with multiple skin neurofibromas or other neurogenic tumors, such as nodular plexiform neurinoma or cerebrospinal tumor. Gastrointestinal stromal tumors are often complicated in patients with neurofibromatosis type 1, although involvement of the appendix is rare, and there have been few reports of appendiceal ganglioneuroma.

**Case presentation:**

The patient was a 29-year-old man diagnosed with neurofibromatosis type 1 based on physical findings and his family history. During the follow-up of neurofibromatosis, computed tomography was performed to detect neurological tumors, such as neurofibromas in the brain, spinal cord, and gastrointestinal tract. Computed tomography showed a markedly thickened appendix wall, and an appendiceal tumor was suspected. Laparoscopic appendectomy was performed, and a 50 × 35 mm appendiceal submucosal tumor was resected with a negative resection margin. At histopathological examination, the tumor was diagnosed as ganglioneuroma; it showed short spindle-shaped cells and ganglion cells diffusely infiltrated into the proper muscle layer and fibrous tissue that grew around nerve cells. The patient was discharged on the 5th postoperative day without postoperative complications and was doing well at 13 months following the operation.

**Conclusions:**

Gastrointestinal stromal tumor and neurofibroma are the most common gastrointestinal tumors associated with neurofibromatosis type 1, but ganglioneuroma of the appendix is rare. Appendiceal neurogenic tumors should be considered in patients with neurofibromatosis type 1, and surgical resection is necessary because of the risk of malignancy.

## Background

Neurofibromatosis is an autosomal dominant inherited disorder and is classified as neurofibromatosis type 1 (NF1), which accounts for 96–97%, or neurofibromatosis type 2 (NF2), which accounts for 3–4% of cases [1, 2]. NF1 is characterized by cutaneous findings, such as multiple neurofibromas and café-au-lait spots, and NF2 is associated with bilateral acoustic neuroma or other nerve tumors. Twenty-five percent of NF1 patients may have gastric and intestinal complications [3]. Regarding the gastrointestinal involvement in NF1, the most common sites are the jejunum (43.6%) and stomach (41.0%); however, the colon and mesentery each accounted for only one of the 39 cases (2.6%) in a previous report [4]. The histology of these lesions is commonly neurofibroma or gastrointestinal stromal tumor (GIST) [5], and ganglioneuroma in the appendix is extremely rare. Here, we report a case of ganglioneuroma of the appendix associated with NF1.

## Case presentation

**Fig. 1:**
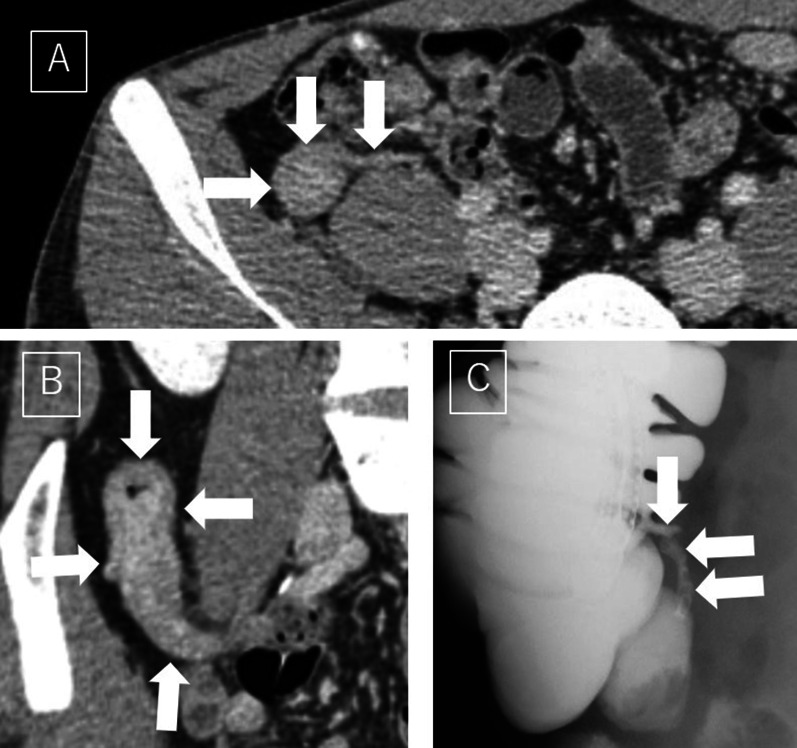
**A**, **B** Enhanced CT revealed an enlarged and diffusely thickened appendix (arrows) on the dorsal side of the ascending colon. The thickened wall was deeply stained with a slight delay. **C** Barium enema image showed no obvious abnormal findings, such as dilation of the appendix (arrows)

The patient was a 29-year-old man. He had pigment spots at birth and neurofibromas all over the skin. His father also had cutaneous neurofibromatosis, and NF1 was diagnosed according to skin findings and his family history at the age of 28. During follow-up of NF1, systemic computed tomography (CT) was performed to detect neurological tumors, such as neurofibromas in the brain, spinal cord, and gastrointestinal tract. The CT scan showed a swollen appendix, and an appendiceal tumor was suspected. He was referred to our department for diagnostic treatment. He was 165 cm tall and weighed 55 kg. Physical examination revealed multiple neurofibromas and café-au-lait spots on the skin of the trunk and extremities. He did not have any abdominal symptoms. His white blood cell count was 8540/µl, and his CRP level was 0.00 mg/dL, showing no increase in the inflammatory reaction. There were no notable findings in terms of other blood counts, biochemistry, or coagulation in laboratory examinations. The tumor markers were carcinoembryonic antigen (CEA) 1.1 U/mL, carbohydrate antigen 19–9 (CA19-9) 5.1 U/mL, alpha-fetoprotein (AFP) 3.0 ng/mL, neuron-specific enolase (NSE) 9.0 ng/mL, and pro-gastrin releasing peptide (pro-GRP) 44.3 pg/mL, all within the normal range. A CT scan revealed an enlarged appendix with a diffusely thickened wall. The thickened wall was well enhanced in the delayed phase (Fig. 1A, B). There were no other findings in the abdominal cavity that suggested malignancy **t**umor or enlarged lymph nodes. Other than neurofibromatosis in the skin, there were no neurological tumors in the brain, spinal cord, or any other part of the body. Colonoscopy revealed hyperplasia of lymphatic tissue of the terminal ileum, and no obvious findings were found at the orifice of the appendix. Barium enema imaging also showed no obvious abnormal findings, such as dilation of the appendix (Fig. 1C).

**Fig. 2:**
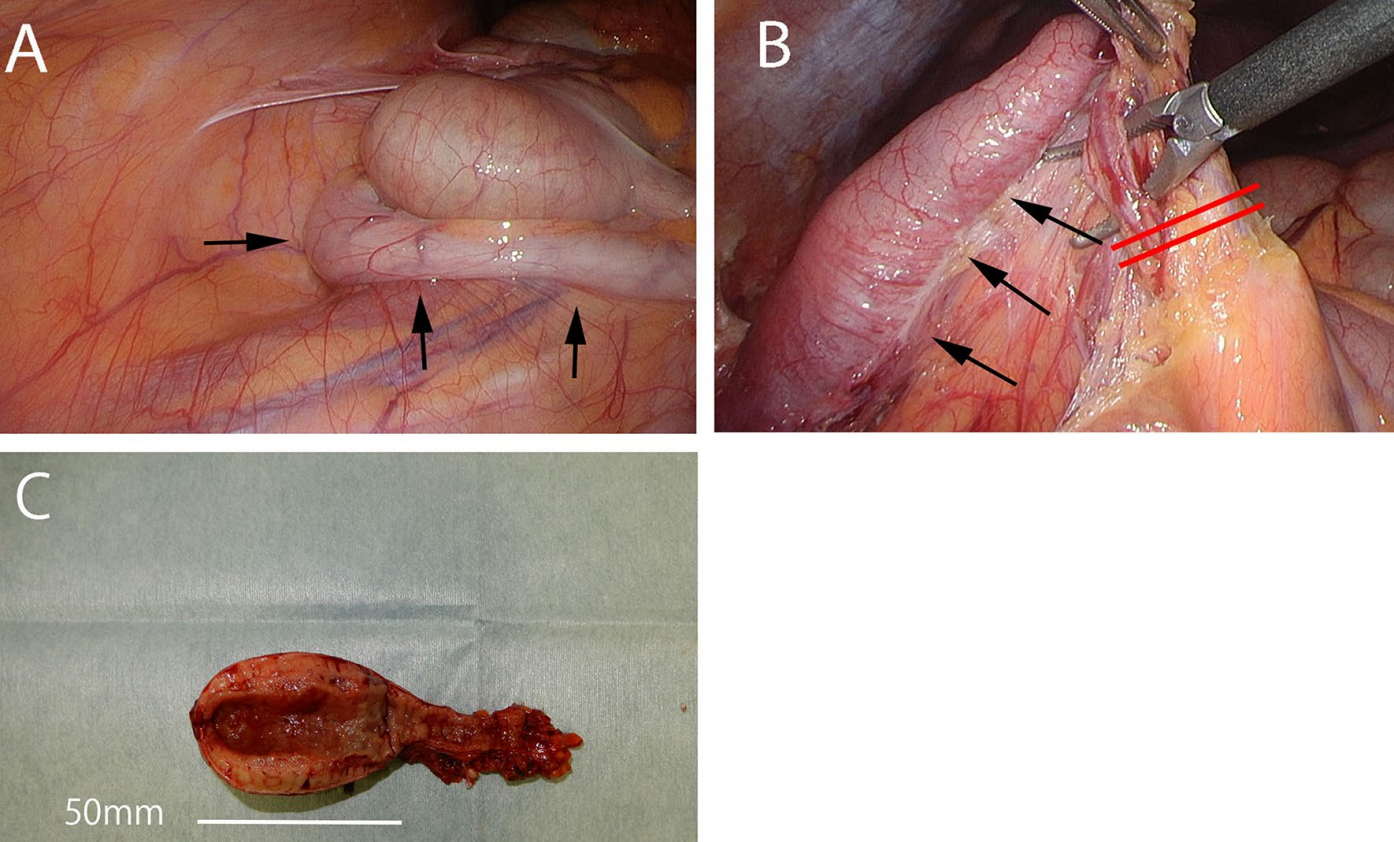
**A**, **B** Appendix was located on the dorsal side of the cecum and was swollen (arrows). The appendix was cut off at the cecum with a linear stapler (red lines). **C** Extracted specimen showed thickening of the wall of the appendix

**Fig. 3:**
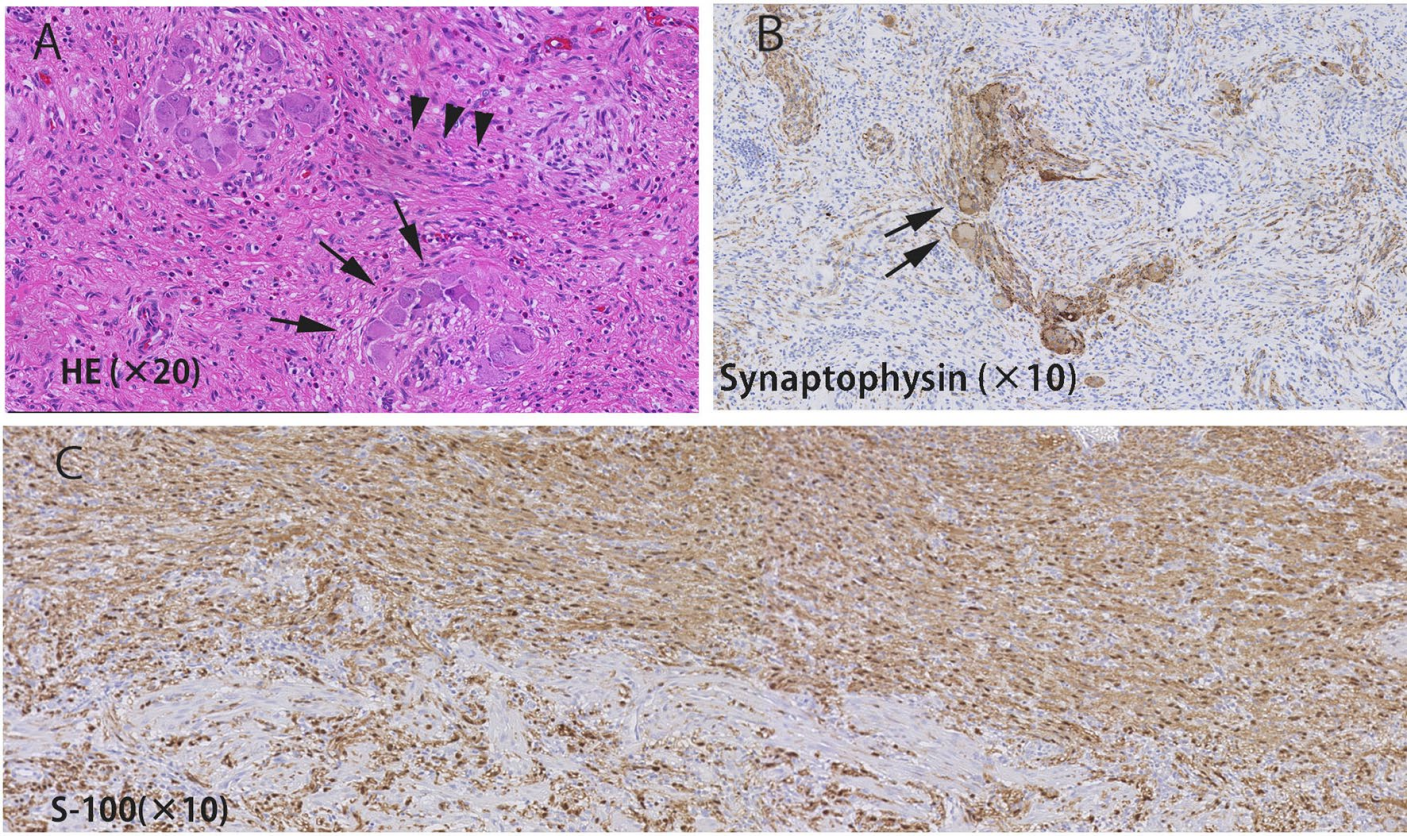
**A** Short spindle-shaped cells (neuron component: arrow heads) and round-shaped cell (ganglion cell: arrows) diffusely infiltrated the proper muscle layer, and fibrous tissue had grown around the nerve cells. **B**, **C** According to immunostaining, ganglion cells were positive for both S-100 **C** and synaptophysin (**B**), and neurons were positive for S-100 only

Then, we performed laparoscopic appendectomy according to the diagnosis of an appendiceal tumor. Under general anesthesia, three trocars were placed, and a pneumoperitoneum was made with 8 cm H_2_O. The markedly swollen appendix was located on the dorsal side of the cecum (Fig. 2A, B). There was no adhesion or dissemination around the tumor. The wall of the cecum was excised with a linear stapler at a sufficient margin from the root of the appendix. Intraoperative diagnosis with frozen sections showed that there was no malignant epithelial tumor, and the resected margin was negative. Then, the operation was finished without lymphadenectomy. Macroscopic findings showed that the tumor was 50 × 35 mm in size and had thick solid white tissue on the cut surface (Fig. 2C, D). Histopathological examination showed short spindle-shaped cells (neuron component) and round-shaped cells (ganglion cells) diffusely infiltrated into the proper muscle layer, and fibrous tissue had grown around the nerve cells (Fig. 3A). According to the immunostaining results, the ganglion cells were positive for both S-100 and synaptophysin, and the neurons were positive for S-100 only (Fig. 3B, C). Pathologically, the cell population was a mix of ganglion cells and neurons, so the finding was ganglioneuroma.

His postoperative course was good, and he was discharged on the 5th postoperative day. He was doing well at 13 months after the operation.

## Discussion

NF1 is an autosomal dominant disease caused by a mutation in the *NF1* gene in the long arm of chromosome 17 (17q11.2); it has an incidence of approximately 1 in 3000 people. *NF1* gene mutations are thought to regulate Ras activity and lead to increased cell growth [6, 7]. Multiple café-au-lait spots and neurofibromas on the skin are typical symptoms of NF1, and neurogenic tumors in various organs throughout the body can also develop. Gastrointestinal tumors associated with neurofibromatosis have been suggested to be common; for example, Davis, Berk and colleagues reported that the prevalence of gastrointestinal tumors with neurofibromatosis was approximately 25% [3]. The appendix is one of the least affected areas of the gastrointestinal tract. One of the largest studies on this topic, conducted by Hochberg et al. evaluated 39 NF1 patients with known gastrointestinal tumors [4] and reported that the most common sites were the jejunum (43.6%) and stomach (41.0%). The colon and mesentery each accounted for only one of the 39 cases (2.6%). Although there are many reports on gastrointestinal tumors associated with NF1, there are few cases of appendiceal tumors. We identified and analyzed 12 case reports of appendiceal tumor associated with NF1 reported through an extensive search using PubMed [8–19]. These are summarized in Table 1. Most of them are reports of GIST and neurofibroma. Bakker et al. examined 61 cases of noncarcinoid gastrointestinal neoplasms with NF1; neurofibromas accounted for 52% of cases, and GISTs accounted for 7% of cases [5]. Other reports showed that the frequency of GISTs in NF1 patients was 6 to 7% [20, 21]. Ganglioneuroma is known to be associated with NF1, but it tends to occur in the adrenal gland and rarely in polypoid form in the gastrointestinal tract [22]. Although several cases of diffuse intestinal ganglioneuromatosis associated with NF1 have been reported [23], the involvement of the appendix is very rarely reported. To the best of our knowledge, this is the second report of appendiceal ganglioneuromas in NF1 next to the first report by Lockhart et al. [12]. Esterson et al. reported a case of appendiceal ganglioneuroma in NF2 [24]. Tumors of the gastrointestinal tract associated with neurofibromatosis type 2 are extremely rare, with no previous reports of appendiceal neurofibromas or GISTs, and only one report of this appendiceal ganglioneuroma. Neurogenic tumors of the gastrointestinal tract are generally asymptomatic, although some patients experience dyspepsia, abdominal pain, anemia, melena, hematemesis, constipation, intestinal obstruction, intestinal perforation or bleeding [25]. In addition, gastrointestinal neurogenic neoplasms were reported to cause sarcomatous degeneration in 5 to 15% of cases [26]. In addition, Kulkarni reported a case of neuroblastoma that recurred as a spinal epidural tumor 11 years after surgery for retroperitoneal ganglioneuroma [27]. In the reported cases, metastasis and recurrence occurred after tumor resection, but the patients had survived for 10 years or more at the time of reporting. In Komo’s report, patients survived 7 months after surgery [18], and many others had long-term survival.

**Table 1:** List of reported cases of appendiceal tumor in NF1

No	Author	Year	Age	Gender	Main symptom	Preoperative diagnosis	Surgical procedure	Postoperative diagnosis	Size (cm)
1	Merck and Kindle[8]	1975	24	M	Abdominal pain	Appendicitis	Appendectomy	Neurofibromatosis	NA
2	Olsen[9]	1987	24	M	Abdominal pain	NA	Appendectomy	Neurofibroma	7 × 3
3	Samuel et al.[10]	1997	19	M	Abdominal pain	Appendicitis	Appendectomy	Neurofibromatosis	3 × 7 × 8
4	Eeden[11]	2000	47	M	Abdominal pain	Acute appendicitis	Appendectomy	Gangliocytic paraganglioma	20
5	Lockhert et al.[12]	2000	33	F	Abdominal pain	NA	Partial right colon resection	Ganglioneuroma	15 × 3
6	Rosenberg et al.[13]	2006	33	F	Asymptom	NA	Appendectomy	Neuroma	12
7	Agaimy et al.[14]	2010	45	M	NA	NA	Appendectomy	NA	0.3
8	Guo et al.[15]	2014	62	F	Abdominal pain	Fallopian tube tumor	Right hemicolectomy	Neurofibroma	9 × 7
9	Jeong et al.[16]	2014	61	M	Abdominal pain	Appendiceal mass	Partial cecum resection	Neurofibroma	NA
10	Ozaki et al. [17]	2015	51	M	Abdominal pain	Apendicitis	Appendectomy	Neurofibroma	3.5 × 2.5 × 2.5
11	Komo et al.[18]	2018	62	F	Asymptom	Cured appendicitis	Cecectomy	Neurofibroma	1.7 × 7
12	Steen et al.[19]	2020	74	M	Abdominal pain	Chronic appendicitis	Appendectomy	Neurofibroma	NA
13	Present case	2020	29	M	Asymptom	Appendiceal tumor	Appendectomy	Ganglioneuroma	5 × 3.5

*NA* not available

The differential diagnosis of an appendiceal tumor includes adenocarcinomas, mucinous neoplasms, carcinoid tumors, lymphomas, and GISTs. Some reports also revealed that patients with NF1 had a risk of malignant tumors of the peripheral nerves [28] and other sarcomas or carcinomas [21]. However, preoperative diagnosis of appendiceal tumor associated with NF1 is difficult, because tumors are rarely exposed through the appendiceal orifice and it is difficult to obtain pathologically diagnosable tissue by colonoscopy. Therefore, surgical resection for diagnostic treatment is often used for appendiceal tumors, as in our case. To the best of our knowledge, unlike retroperitoneal ganglioneuroma, there have been no reports about NF1-related gastrointestinal tract tumors with lymph node metastasis. Therefore, we believe that local resection of the tumor is basically sufficient and that lymph node dissection is not necessary.

## Conclusions

Here, we report a rare case of ganglioneuroma of the appendix pointed out during follow-up of NF1. Appendiceal tumors associated with NF1 are potentially malignant, and surgical resection should be considered for diagnostic treatment.

## Data Availability

All data generated or analyzed during this study are included in this published article.

## Abbreviations

NF1: Neurofibromatosis type 1
NF2: Neurofibromatosis type 2
GIST: Gastrointestinal stromal tumors
CT: Computed tomography
